# Supplementary material for: Differences in auditory brainstem responses between laboratory-reared and wild-caught prairie voles (Microtus ochrogaster)
Source: PLoS One. 2026 Feb 20;21(2):e0335120. doi: 10.1371/journal.pone.0335120 (PMC12923069; doi:10.1371/journal.pone.0335120)
Supplement: S1 File — Tables supporting audiogram data for males and females. (DOCX) [file pone.0335120.s001.docx]

**Electronic Supplementary Material**

**Differences in Auditory Brainstem Responses between Laboratory-reared and Wild-caught Prairie Voles (*Microtus ochrogaster*)**

Luberson Joseph, Naleyshka Colon-Rivera, Emily M. New, Desi M. Joseph, Jessica A. Hurd, Casey E. Sergott, Elizabeth A. McCullagh

Corresponding author:

Elizabeth A. McCullagh

E-mail: [elizabeth.mccullagh@okstate.edu](mailto:elizabeth.mccullagh@okstate.edu)

Department of Biology, Oklahoma State University (OSU), College of Arts and Sciences, Stillwater, Oklahoma 74078, USA

**Audiogram Female data only:**

| **Frequency (kHz)** | **Laboratory-reared**  **mean ± S. E** | **Wild-caught mean ± S. E** | **t-ratio** | **p-value** |
| --- | --- | --- | --- | --- |
| **1** | 63.5 ± 2.61 | 53.0 ± 3.43 | 2.435 | 0.015 |
| **2** | 49.7 ± 2.63 | 46.1 ± 3.47 | 0.839 | 0.402 |
| **4** | 43.2 ± 2.63 | 34.1 ± 3.47 | 2.109 | 0.036 |
| **8** | 31.5 ± 2.63 | 20.1 ± 3.47 | 2.622 | 0.009 |
| **16** | 32.6 ± 2.63 | 21.1 ± 3.47 | 2.663 | 0.008 |
| **24** | 33.2 ± 2.63 | 28.1 ± 3.47 | 1.190 | 0.235 |
| **32** | 29.1 ± 2.70 | 35.6 ± 3.47 | -1.472 | 0.142 |
| **46** | 41.6 ± 2.70 | 43.1 ± 3.47 | -0.335 | 0.738 |

**Table S1:** Statistical measures of auditory brainstem response thresholds between female laboratory-reared (N = 17) and wild-caught (N= 10) prairie voles. Values displayed represent the mean at each frequency tested ± standard error, the t-ratio value, and the p-value of ABR response thresholds between laboratory-reared and wild-caught prairie voles.

**Audiogram Male data only:**

| **Frequency (kHz)** | **Laboratory-reared**  **mean ± S. E** | **Wild-caught mean ± S. E** | **t-ratio** | **p-value** |
| --- | --- | --- | --- | --- |
| **1** | 59.7 ± 3.62 | 55.0 ± 4.56 | 0.805 | 0.422 |
| **2** | 50.2 ± 3.71 | 46.1 ± 4.56 | 0.687 | 0.493 |
| **4** | 47.8 ± 3.71 | 33.3 ± 4.56 | 2.466 | 0.015 |
| **8** | 31.5 ± 3.70 | 17.2 ± 4.56 | 2.423 | 0.017 |
| **16** | 31.3 ± 3.52 | 19.2 ± 4.56 | 2.113 | 0.037 |
| **24** | 34.0 ± 3.52 | 23.9 ± 4.56 | 1.757 | 0.082 |
| **32** | 30.7 ± 3.52 | 38.3 ± 4.56 | -1.4327 | 0.187 |
| **46** | 37.2 ± 3.62 | 38.9 ± 4.56 | -0.292 | 0.771 |

**Table S2:** Statistical measures of auditory brainstem response thresholds between male laboratory-reared (N = 16) and wild-caught (N= 10) prairie voles. Values displayed represent the mean at each frequency tested ± standard error, the t-ratio value, and the p-value of ABR response thresholds between laboratory-reared and wild-caught prairie voles.
